# *ZmHPAT2* Regulates Maize Growth and Development and Mycorrhizal Symbiosis

**DOI:** 10.3390/plants14101438

**Published:** 2025-05-11

**Authors:** Kailing Xie, Guoqing Wang, Ying Ni, Minghui Shi, Lixue Sun, Beijiu Cheng, Xiaoyu Li

**Affiliations:** Key Laboratory of Crop Stress Resistance and High-Quality Biology of Anhui Province, Anhui Agricultural University, Hefei 230036, China; 15056907002@163.com (K.X.); w2138273756@163.com (G.W.); 15215517537@163.com (Y.N.); 18905619514@163.com (M.S.); 23720594@stu.ahau.edu.cn (L.S.)

**Keywords:** maize, *ZmHPAT2*, vegetative growth, AM fungal colonization

## Abstract

Hydroxyproline O-arabinosyltransferase (HPAT), a critical enzyme in plant glycosylation pathways, catalyzes the transfer of arabinose to the hydroxyl group of hydroxyproline residues. This enzyme contains a canonical GT95 glycosyltransferase, a structural hallmark of this carbohydrate-active enzyme family. HPAT mediates arabinosylation of diverse cellular targets, including cell wall extension and small signaling peptides. Emerging evidence has shown that HPAT orthologs regulate plant development and symbiotic interactions through post-translational modification of *CLV1*/LRR Extracellular (CLE) peptides. Although the molecular functions of *HPAT* genes have been characterized in model plants such as *Arabidopsis thaliana* and *Lotus japonicus*, their roles remain unexplored in *Zea mays* L. In this study, we used *ZmHPAT2* homozygous mutants to explore the function of the maize *HPAT* gene. Sequence analysis identified a N-terminal signal peptide targeting the Golgi apparatus and promoter elements responsive to AM fungal colonization. Phenotypic analysis revealed its negative regulatory role: *zmhpat2* promotes vegetative growth (increased plant height and accelerated flowering) and enhances AM symbiosis (increased colonization rate). Mechanistic studies demonstrated that *ZmHPAT2* possesses dual regulatory functions—the activation of auxin signaling and repression of *ZmMYB1*-mediated arbuscular degradation pathways. In addition, overexpression of *ZmHPAT2* in *Lotus japonicus* inhibits growth (reduced plant height) and impairs symbiotic interactions. Our findings establish *ZmHPAT2* as a critical node to regulate auxin and symbiotic signaling, providing novel insights into plant glycosylation-mediated development. This work not only advances our understanding of maize growth regulation but also identifies potential targets for crop improvement through arabinosylation pathway manipulation.

## 1. Introduction

Maize is one of the most consumed crops in the world and is the main food crop in China [1]. Maize (*Zea mays* L.), a thermophilic C4 cereal crop with high photosynthetic efficiency, possesses a functionally sophisticated root system that is crucial for growth, development, and stress responses [2]. Root systems primarily facilitate the acquisition of water and nutrients from the soil [3]. Furthermore, as the primary interface for plant–soil interactions, the root system plays a pivotal role in mediating plant responses to environmental fluctuations, thereby influencing crucial agronomic traits [4]. These traits include drought tolerance [5,6], flood resistance, lodging resistance [7,8], and nutrient utilization efficiency through symbiotic associations with arbuscular mycorrhizal fungi in the rhizosphere [6]. The symbiotic relationship between arbuscular mycorrhizal (AM) and maize represents a mutualistic association characterized by reciprocal nutrient exchange. AM fungi enhance the host plant’s capacity for water and mineral nutrient acquisition from the soil, while simultaneously improving its stress tolerance. In return, the fungal partner receives fixed carbon compounds from the host plant. Both sides exchange these substances through highly branched arbuscular structures in plant cells [9,10]. In addition, plant height is an important trait and is closely related to the lodging resistance of plants and the yield of some cash crops [11]. Plant height is determined by stem cell division, expansion, and differentiation [12,13]. Plant hormones are important regulators of plant height, such as gibberellin (GA), brassinosteroid (BR), cytokinin (CK), and auxin (IAA) [14,15]. The regulation process of plant height is very complex, and phytohormone-related pathways have a very important impact on plant height [16].

Hydroxyproline O-arabinosyltransferase (HPAT) is a key enzyme that catalyzes the conversion of arabinosyltransferase to hydroxyproline. It is a small, highly conserved family of plant-specific glycosyltransferases, belonging to the GT95 glycosyltransferase family [17]. This family adds arabinose to a variety of proteins, including cell wall-associated extension proteins and small signal peptides [18]. In *Arabidopsis thaliana*, the *hpat1-1/hpat2-1* double mutant has been shown to exhibit various phenotypes such as longer hypocotyls than the wild type, reduced cell wall thickness, early flowering [14,15], and decreased chlorophyll content. In addition, the pollen development ability of the *Arabidopsis thaliana* double mutant *hpat1/hpat3* was lower than of that the wild type [19]. When grown on in vitro pollen germination medium, the pollen tubes of the double mutant *hpat1/hpat3* showed many defects, including reduced length of pollen tube, increased frequency of pollen tube rupture, and increased width of pollen tube. The *FIN4* gene of the tomato HPAT gene family was expressed in developing pollen, and the *fin4* mutant with a missing catalytic domain showed a decreased pollen tube germination rate and impaired pollen hydration. Moreover, legumes have evolved mechanisms to maintain a symbiotic balance between nodule formation, which fulfills their nitrogen requirements, and the energy levels necessary for other biological processes [20]. These mechanisms are known as the autoregulation of neoplasia (AON) pathway. *LjPLENTY* and *MtRDN3 (Medicago truncatula ROOT DETERMINED NODULATION3)* all belong to the HPAT gene family and may mediate the arabinosylation of *CLV1/*LRR Extracellular (CLE) peptides that they modify, playing a crucial role in the AON of their respective species [21,22]. Studies on the *PLENTY* gene of *Lotus japonicus* showed that the mutant *plenty* had an increased number of nodules and shorter roots under both symbiotic and non-symbiotic conditions [19]. These results suggest that HPAT family genes can play a role in plant development and AM symbiosis by modifying the CLE peptides.

In recent years, HPAT has been reported to have functions related to growth, development, and symbiosis in *Arabidopsis thaliana*, *Lotus japonicus*, and *Solanum lycopersicum* [19,21,23]. However, there have been no reports on the functional study of HPAT in maize. Therefore, exploring the functions of the HPAT gene family in maize is of great significance for maize research and the development of germplasm resources.

## 2. Results

### 2.1. Bioinformatics Analysis of the ZmHPAT2 Gene

**Figure 1:**
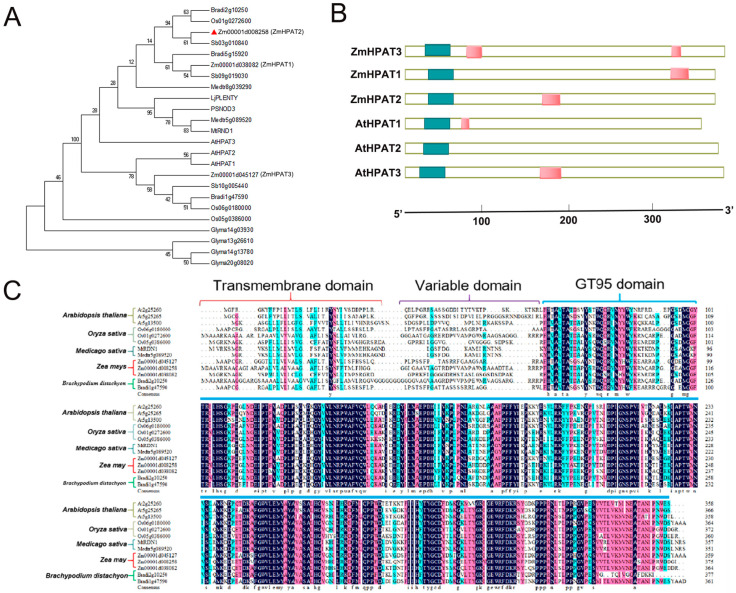
Bioinformatics analysis and characterization of *ZmHPAT2*. (**A**) Evolutionary analysis of the HPAT gene family in maize (*Zea mays*) and other species: *Brachypodium distachyon* (Bd), *Sorghum bicolor* (Sb), *Medicago truncatula* (Mt), *Glycine max* (Gm), *Oryza sativa* (Os), *Arabidopsis thaliana* (At), *Lotus japonicus* (Lj), *and Pisum sativum* (Ps). (**B**) Analysis of the domains of the maize HPAT gene family and the HPAT gene family reported in *Arabidopsis*, green represents the N-terminal conserved region. (**C**) Protein sequence alignment of the maize HPAT gene family and HPAT genes from other species. The red bracketed region represents the transmembrane region, the purple bracketed region represents the variable region, the blue bracket represents the highly conserved catalytic domain of GT95, and the highly conserved amino acids are shown in black.

Three HPAT genes in maize were homologous to those in Arabidopsis, rice, and soybean, and a phylogenetic tree was constructed using MEGA7.0. The results of the evolutionary tree showed that HPAT proteins are a widely existing family in the plant kingdom, including *Salvia verticillata*, *Arabidopsis thaliana*, *Glycine max*, *Medicago sativa*, etc. This widespread distribution suggests the evolutionary conservation of hydroxyproline arabinosylation pathways in plant evolution. Among them, At2g25260 (*AtHPAT2*), At2g25265 (*AtHPAT1*), and At5g13500 (*AtHPAT3*) are three genes that have been previously characterized as regulators of the growth and development of *Arabidopsis thaliana*, whereas the mutant Medtr5g089520 (*MtRDN1*) has been reported to show a highly nodular phenotype [24]. Phylogenetic analysis of the gene family revealed that *ZmHPAT2* is closely related to *LjPLENTY*, *PsNOD3*, and *MtRDN1*, all of which have been reported to be related to fungal symbiosis (Figure 1A).

The domains of the HPAT family genes in maize and three genes in *A. thaliana* were analyzed using the SMART website. It was found that they all contain a domain at the N-terminal (Figure 1B). The HPAT genes of *A. thaliana*, rice, sorghum, and soybean were subjected to protein sequence comparison with those of maize. The comparison results showed that HPAT family genes contained multiple conserved amino acid residues in different species, indicating that HPAT family genes were highly conserved during species evolution (Figure 1C). Comparative analysis using the DANMAN software(version 6.0) revealed that the protein sequence consistency was 69.64%. The protein sequence of *ZmHPAT2* included a transmembrane domain and a highly conserved GT95 catalytic domain, suggesting that *ZmHPAT2* encodes a possible functional HPAT protein.

### 2.2. Subcellular Localization of ZmHPAT2

**Figure 2:**
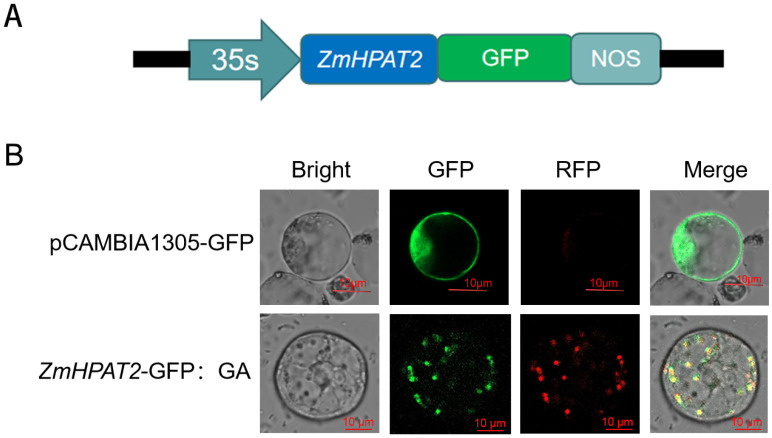
Subcellular Localization of *ZmHPAT2.* (**A**) Schematic diagram of the construction of the subcellular localization vector. (**B**) Subcellular localization of *ZmHPAT2*-GFP in protoplast of maize. Four visual fields were observed: bright channel (Bright), green channel (GFP), red channel (RFP), and merged. Scalebar = 10 µm.

The HPAT gene family belongs to the GT95 glycosyltransferase family and is involved in post-translational modification of proteins. It has been reported that HPAT genes in other species are localized in the Golgi apparatus [17]. To show the localization of *ZmHPAT2*, the 35S::*ZmHPAT2*-GFP fusion vector and maize protoplasts were used to observe subcellular localization (Figure 2A). The constructed fusion vector was transferred into maize protoplasts using a Polyethylene glycol-mediated method. During transfer, the maize Golgi positioning signal plasmid was co-transformed as an indicator. As shown in the figure (Figure 2B), the empty 35S::GFP carrier without gene fusion showed green fluorescence in the nucleus, cell membrane, and cytoplasm, while the 35S::*ZmHPAT2*-GFP carrier showed a spot-like green fluorescence distribution in the cell, which was highly overlapped with the red fluorescence presented by the Golgi localization signal plasmid. Therefore, we determined that *ZmHPAT2* is located in the Golgi apparatus. This is consistent with the function of *ZmHAPT2* in post-translational modification of small peptides.

### 2.3. ZmHPAT2 Positively Regulates Root Development

For subsequent experiments, we identified the homozygous mutant of *ZmHPAT2*. In the mutant detection, by comparing the sequencing results with the wild type, if the mutation site shows a transition from T base to G base and exhibits a single peak, it indicates a homozygous plant; if the mutation site shows a double peak, it indicates a heterozygous plant.

**Figure 3:**
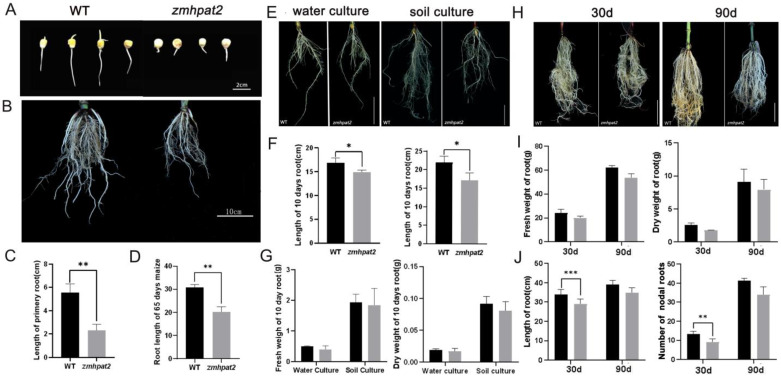
Analysis of the root development characteristics of the *zmhpat2* mutant. (**A**) Root growth phenotype of maize mutants during germination, the scale bar on the image represents 2 cm. (**B**) Field maize root phenotype, with the scale bar representing 10 cm. (**C**) Length of primary root during germination. (**D**) Root length of 65 days maize. (**E**) Early-stage root phenotype of water culture (**left**) and soil culture (**right**), with the scale bar representing 10 cm. (**F**) Water culture (**left**) and soil culture (**right**) maize root length. (**G**) Fresh weight (**left**) and dry weight (**right**) of 10 day maize root. (**H**) Root phenotype of maize at 30 and 90 days of greenhouse cultivation, with the scale bar representing 10 cm. (**I**) Fresh weight (**left**) and dry weight (**right**) of maize roots at 30 and 90 days of greenhouse cultivation. (**J**) Root length of maize at 30 and 90 days (**left**) and Number of nodal roots of maize at 30 and 90 days (**right**). Asterisks indicate significant differences and * represents *p* < 0.1, ** *p* < 0.01 and *** *p* < 0.001. The unmarked represents “ns” (not significant). Statistical analysis was performed using the Student’s *t*-test.

To determine whether *ZmHPAT2* affects maize root development, the homozygous mutant *zmhpat2* was germinated with wild type (WT) as a control. After 4 days, the primary root length of the *zmhpat2* mutant was significantly shorter than that of the wild type (WT), showing a 60% reduction in root length (Figure 3A). Additionally, a sampling analysis of maize plants grown in the field for 65 days revealed that the root length of the *zmhpat2* mutant was still shorter than that of the wild type (Figure 3B). The root length statistical results are consistent with the phenotypes, showing significant differences between the wild-type and mutants (Figure 3C,D).

To further investigate the root growth of the maize mutant *zmhapt2*, full, homozygous seeds of the same size were selected for water and soil culture, and wild-type B73 was used as the control. Sample the corn on the 10th day. The results showed that under hydroponic conditions, the nodal roots length of the mutant *zmhpat2* was significantly shorter than that of the wild type, whereas there was no difference in the number of the fibrous roots (Figure 3E). Similarly, under soil culture conditions, the nodal roots length of mutant *zmhapt2* was still shorter than that of the wild type. In contrast to water cultivation conditions, under soil cultivation, the wild type had a significantly greater number of fibrous roots than the mutant (Figure 3E). This is because soil culture maize faces greater challenges in obtaining water and nutrients compared to water culture, requiring the plants to develop more lateral roots to absorb sufficient nutrients from the soil. The root length and dry weight of the *zmhapt2* mutant and wild type were statistically analyzed under two different cultivation conditions. These results are consistent with the observed phenotypes. Among them, the difference in root length between the two reached a significant level, but there was little difference between dry and fresh weights (Figure 3F,G).

Additionally, to investigate whether the root system of the *zmhpat2* mutant differed from that of the wild type at different stages, maize plants grown in the greenhouse were sampled at the middle and late stages. As shown in Figure 3, both 30-day-old and 90-day-old plants showed the same phenotype as in the earlier stage, and the nodal roots length of mutant *zmhapt2* was shorter than that of wild type (Figure 3H). The statistical analysis of dry and fresh weights, as well as root length, was consistent with the observed phenotype (Figure 3I,J). Furthermore, statistical analysis of the number of nodal roots revealed that the *zmhapt2* mutant had fewer nodal roots than the wild type (Figure 3J).

### 2.4. ZmHPAT2 Negatively Regulated Plant Height and Loose Powder

**Figure 4:**
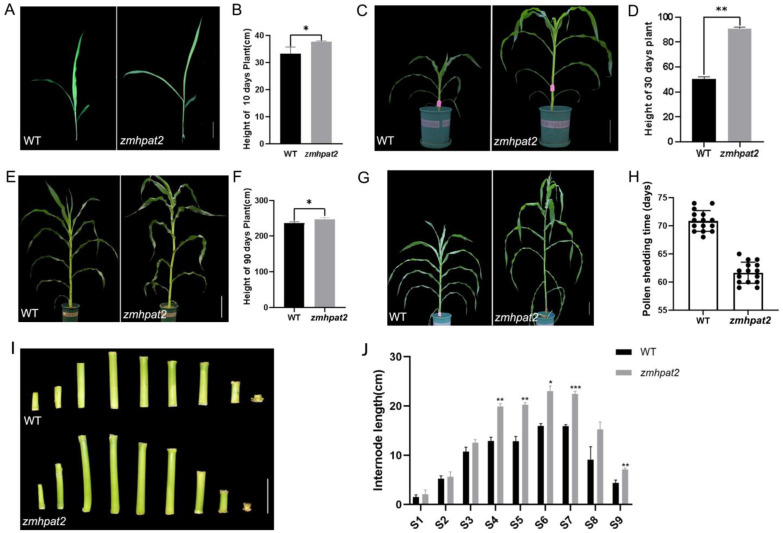
Analysis of the aboveground phenotype of *zmhpat2* mutant plants. (**A**) Three-leaf stage plant growth phenotype, scale bar is 10 cm. (**B**) Height of 10 days plant. (**C**) Phenotype of plants grown in greenhouse for 30 days, scale bar is 10 cm. (**D**) Height of 30 days plant. (**E**) Phenotype of plants grown in greenhouse for 90 days, scale bar is 10 cm. (**F**) Height of 90 days plant. (**G**) Flowering phenotype of the wild type and mutant *zmhpat2*, scale bar is 10 cm. (**H**) Pollen shedding time statistics of the wild type and mutant *zmhpat2*. (**I**) Intercalary phenotype of the wild type and mutant *zmhpat2*, scale bar is 20 cm. (**J**) Statistical analysis of internode length. All the *zmhpat2* are compared with their respective wild types. Asterisks indicate significant differences and * represents *p* < 0.1, ** *p* < 0.01 and *** *p* < 0.001. The unmarked represents “ns” (not significant). Statistical analysis was performed using the Student’s *t*-test.

In addition to the differences in root development, aboveground development of the *zmhpat2* mutant also differed from that of the wild type. The height of the mutant *zmhpat2* was higher than that of the wild type at different stages of maize growth (Figure 4A–F). At the three-leaf stage, it can be observed that the height of the mutant *zmhpat2* plant is higher than that of the wild type, mainly in the distance from the root system to the first leaf (Figure 4A,B). After 30 days of growth in the greenhouse, a significant difference in plant height was observed between the *zmhpat2* mutant and the wild type (Figure 4C). Statistical analysis showed that this difference was highly significant (Figure 4D). The same phenotype was observed after 90 days of growth in the greenhouse (Figure 4E,F). When the mutant *zmhpat2* grew to 65 days in the field, the plant height was also greater than that of the wild type (Figure 4G). Interestingly, as shown in Figure 4H, we noticed that the pollen shedding time of the mutant *zmhpat2* was 9 to 10 days earlier than that of the wild type.

In addition, according to the statistics of internode lengths of the plant stems (Figure 4I,J), the height of the mutant plant was mainly due to the elongation of the internodes. The first internode above the ground was designated as S1, and the statistical data showed significant differences in the lengths of the internodes between S4 and S7. These results indicate that *ZmHPAT2* plays a negative role in the height development of maize plants, and the mutant *zmhpat2* showed a higher plant height than the wild type.

These results indicate that *ZmHPAT2* may play a regulatory role in maize growth and development but may play a different role in maize plant height and root development.

### 2.5. ZmHPAT2 Is Induced to Express by AM Fungi

**Figure 5:**
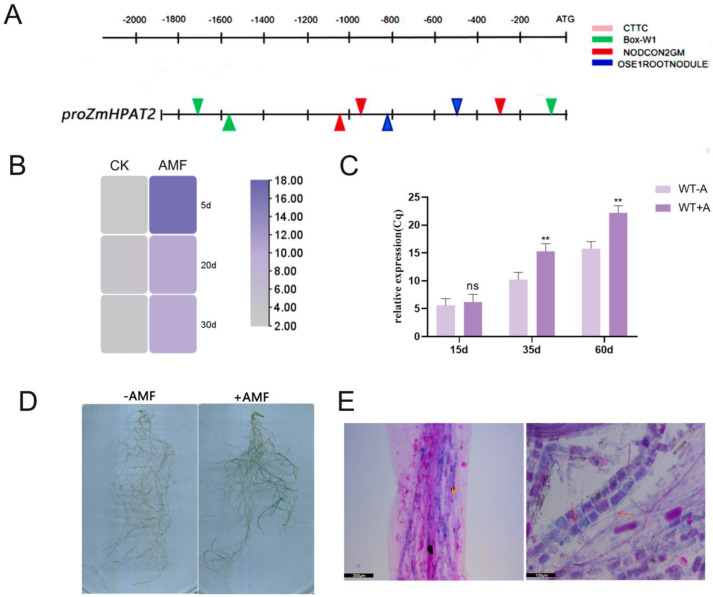
Analysis of mycorrhizal-induced *ZmHPAT2* expression. (**A**) Analysis of AM fungi symbiosis regulatory elements in the promoters of *ZmHPAT2*. (**B**) Heatmap of the *ZmHPAT2* gene expression induced by AMF at different stages. (**C**) The relative expression level of *ZmHPAT2* in maize roots inoculated with AMF and non-inoculated controls measured was by qRT-PCR; 15d, 35d, 60d, respectively, represent the wild-type B73 and AM symbiosis at 15 days, 35 days, and 60 days; WT+A represent AM fungi-inoculated wild-type plants; WT-A represent non-inoculated wild-type controls. (**D**) Detection of GUS expression by p*ZmHPAT2* induced by AM fungi; −AMF indicates non-inoculation with arbuscular mycorrhizal fungi, and +AMF denotes inoculation with AM fungi. (**E**) Local microscopic magnification of roots with fuchsine. The arrow in the left image indicates the overlap of GUS expression with the red arbuscular structures, with a scale bar of 200 µm. The arrow in the right image points to the purple arbuscular structures formed around the vascular bundles, with a scale bar of 100 µm. Asterisks indicate significant differences: ** represents *p* < 0.01; "ns" indicating no significant difference, statistical method used was Student’s *t*-test.

The promoter regions of genes induced by AM generally contain cis-acting elements related to mycorrhizal induction. Previous studies have identified four acting elements, W-BOX, CTTC, NODCON2GM, and OSEROOTNODULE, which are distributed in the promoter regions of genes induced by rhizobia and AM fungi [25,26]. The promoter of this gene was analyzed and mapped online using the RAST website. The results showed that the promoter region of *ZmHPAT2* contained the aforementioned symbiosis-related acting elements (Figure 5A).

Simultaneously, based on the symbiotic transcriptomic data, we constructed a heatmap and found that *ZmHPAT2* was upregulated by AM to varying degrees during different stages of symbiosis (Figure 5B). Wild-type maize B73 was symbiotic with AM fungi and samples were collected at different times for quantitative analysis. It was found that the expression of *ZmHPAT2* was significantly upregulated at different stages after inoculation with the fungus compared to the control, especially in the mid and late stages of symbiosis (Figure 5C).

To further determine whether *ZmHPAT2* is induced by AM fungi, we constructed the p*ZmHPAT2*-GUS vector and transformed it into *L. japonicus* using *Agrobacterium tumefaciens* to obtain hairy roots with the fusion plasmid. The successfully transformed hairy roots were inoculated with AM fungi, and the expression of the GUS gene was detected to determine whether the target gene was induced by AM fungi. Two groups of experiments were set up: one group of transformed hairy roots was inoculated with AM fungus and the other group was treated normally as a control. As seen in the figure, the transformed hairy roots inoculated with AM fungi showed an obvious blue color. (Figure 5D).

After GUS staining, the root segments were counterstained using a magenta staining solution. The root segments were selected for preparation until the decolorization was complete. GUS expression and symbiosis in root segments were observed by microscopy. More purple arbuscular structures formed around the vascular bundles (Figure 5E). Under high magnification, GUS expression can be seen to overlap with red arbuscular structures as well as some individual arbuscular structures. This suggests that *ZmHPAT2* may regulate symbiosis by influencing the arbuscular structure.

### 2.6. ZmHPAT2 Negatively Regulates AM Fungi Symbiosis in Maize

To study the effect of *ZmHPAT2* on the symbiosis between maize and AM fungi, the mutant *zmhapt2* was inoculated with AM fungi, while the wild-type inoculated AM fungi were used as controls. They were co-cultivated in the greenhouse and sampled in batches at 5, 35, and 60 d.

**Figure 6:**
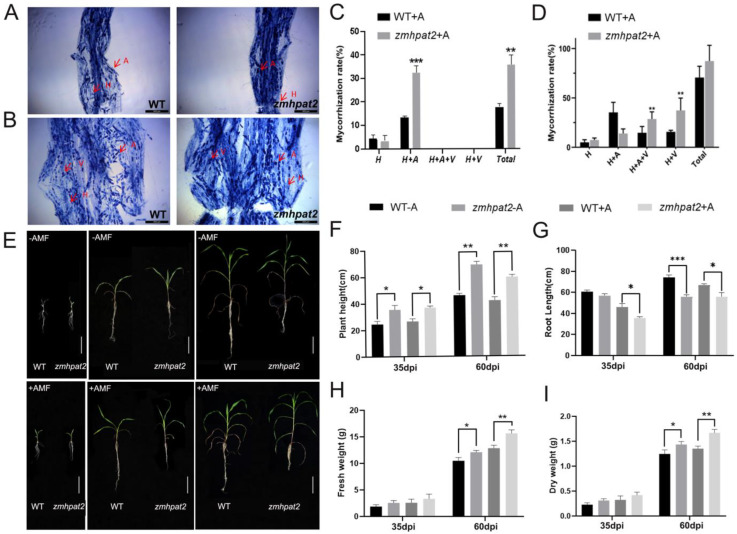
*zmhpat2* mutant and AM fungi symbiotic effect analysis. (**A**) Trypan blue staining of mycorrhizal roots at 35 days of symbiosis. (**B**) Trypan blue staining of mycorrhizal roots at 60 days of symbiosis. (**C**) Mycorrhizal colonization rate statistics at 35 days of symbiosis. (**D**) Mycorrhizal colonization rate statistics at 60 days of symbiosis, H: Hyphae; A: Arbuscule; V: Vesicle. (**E**) Phenotypes of wild-type and *zmhpat2* mutant before and after symbiosis with AM fungi. The scale bar is 20 cm. (**F**) Plant height at 30 and 60 days of symbiosis. (**G**) Root length at 30 and 60 days of symbiosis. (**H**) Fresh weight at 30 and 60 days of symbiosis. (**I**) Dry weight at 30 and 60 days of symbiosis. The +A and −A *zmhpat2* are compared with their respective wild types. Asterisks indicate significant differences and * represents *p* < 0.1, ** *p* < 0.01 and *** *p* < 0.001. The unmarked represents “ns” (not significant). Statistical analysis was performed using the Student’s *t*-test.

Microscopic analysis of mycorrhizal staining revealed that, 35 days post-inoculation (dpi), a functional symbiotic relationship was established between arbuscular mycorrhizal (AM) fungi and host plants, as evidenced by the presence of extensive hyphal networks and well-developed arbuscular structures within the root cortex (Figure 6A). After 60 days of symbiosis, the total colonization rate of the *zmhapt2* mutant significantly increased, and it was markedly higher than that of the wild type (Figure 6B). Specific statistics showed that at 35 dpi, the colonization rate of the *zmhapt2* mutant was higher than that of the wild type, with the symbiosis rate of the *zmhapt2* mutant being 32%, whereas that of the wild type was only 13% (Figure 6C). In terms of fungal symbiosis, the hyphal and arbuscular structures (H+A) of the zmhapt2 mutant were significantly higher than those of the wild type. After 60 days of symbiosis, the *zmhapt2* mutant contained approximately 43.6% hyphal and vesicular structures (H+V) and 32.3% hyphal, arbuscular, and vesicular structures (H+A+V), while the wild type contained only 21% hyphal and vesicular structures (H+V) and 15.3% hyphal, arbuscular, and vesicular structures (H+V+A) (Figure 6D). In contrast to the symbiosis at 35 days, the *zmhapt2* mutant had fewer arbuscular structures at this point than the wild type. During the later stages of symbiosis, the arbuscular structures began to degrade and form vesicular structures.

Next, from the perspective of growth phenotype, the growth of wild-type and mutant *zmhapt2* plants at 5 days after inoculation was similar to that of control plants. After 35 and 60 days of fungal inoculation, the aboveground parts of the experimental group maize plants were more robust, whereas the roots were slightly shorter than those of the control group (Figure 6E). Maize plants in different periods were sampled, and their root length, plant height, and dry and fresh weights were measured.

From the statistical results of plant height, the height of the mutant *zmhpat2* was greater than that of the wild type, regardless of whether the plants were inoculated with fungi (Figure 6F). This further supports the role of the *ZmHPAT2* gene in negatively regulating plant height development in maize, leading to the mutant *zmhpat2* exhibiting a taller plant height than the wild type. Interestingly, after 60 days of symbiosis, the height of the non-symbiotic plants was significantly higher than that of the mycorrhizal plants, but their stem diameter was noticeably lower than that of the mycorrhizal plants. (Figure 6E,F).

According to the statistical results of root length, the root length of the mutant *zmhpat2* was shorter than that of the wild type under all conditions (Figure 6G). This further confirmed that *ZmHPAT2* positively regulated maize root elongation. In addition, the root length of symbiotic plants was shorter than that of non-symbiotic plants at both 30 and 60 d of symbiosis (Figure 6G). This is mainly because under conditions of nutrient deficiency, the root systems of non-symbiotic plants can only expand downward to absorb the nutrients needed for growth, while the root systems of symbiotic plants form a symbiotic relationship with AM fungi through well-developed lateral roots, and nutrients are delivered to them by AM fungi [27,28].

The dry and fresh weight statistics showed that symbiotic plants outperformed non-symbiotic plants at all stages, and the mutant *zmhpat2* grew better than the wild type (Figure 6H). This is mainly because, on the one hand, the *ZmHPAT2* gene negatively regulates plant height, and on the other hand, the symbiotic rate of the mutant *zmhpat2* is higher than that of the wild type, resulting in stronger plant growth.

These results indicate that *ZmHPAT2* plays a negative regulatory role in the symbiosis between maize and AM.

### 2.7. Overexpression of ZmHPAT2 Inhibits Plant Height and Mycorrhizal Symbiosis

To further study the effects of *ZmHPAT2* on plant growth, development, and symbiosis regulation, a fusion vector overexpressing the ZmHPAT2 gene was constructed. *ZmHPAT2* overexpression plants were obtained by *Agrobacterium tumefaciens* LBA9402, which was transferred into the hairy roots of *L. japonicus*. The overexpressed plants were transferred from the medium into the culture medium and watered with a low-phosphorus nutrient solution once a week. The plants were sampled and analyzed after eight weeks of culture. RNA from wild-type MG20 and overexpressing strains was extracted, and RT-PCR was used to verify the expression of *ZmHPAT2* in transgenic plants. The results showed that the expression of *the ZmHPAT2* gene in transgenic plants was significantly upregulated compared to the wild type (Figure S2).

**Figure 7:**
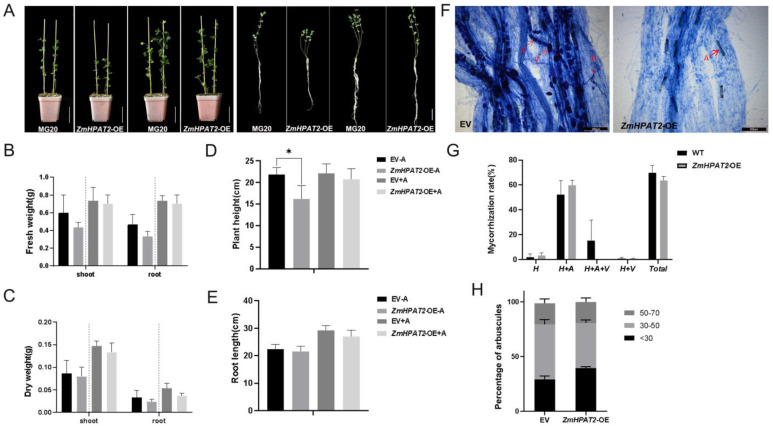
Functional analysis of *ZmHPAT2* the overexpression plants. (**A**) Phenotypes of *ZmHPAT2* overexpression transgenic *L. japonicus* symbiotic and non-symbiotic with AM fungi. (**B**) Fresh weight statistic of symbiotic and non-symbiotic plants. (**C**) Dry weight statistic of symbiotic and non-symbiotic plants. (**D**) Plant height of symbiotic and non-symbiotic plants. (**E**) Root length of symbiotic and non-symbiotic plants; EV-A represents MG20 without AM fungus inoculation, while *ZmHPAT2*-OE-A refers to the overexpressed *ZmHPAT2 L. japonicus* roots without AM fungus inoculation; EV+A represents MG20 with AM fungus inoculation, and *ZmHPAT2*-OE+A refers to the overexpressed *ZmHPAT2 L. japonicus* roots with AM fungus inoculation. (**F**) Trypan blue staining of mycorrhizal roots in control and *ZmHPAT2* overexpression transgenic plants. (**G**) Mycorrhizal colonization rate statistics in symbiotic plants. (**H**) Percentage of different arbuscule sizes in symbiotic plants. H: Hyphae; A: Arbuscule; V: Vesicles; <30 indicates arbuscule size smaller than 30 μm, 30–50 represents arbuscule sizes ranging between 30 and 50 μm, 50–70 represents arbuscule sizes ranging between 50 and 70 μm. The +A and −A *ZmHPAT2-OE* are compared with their respective EV. Asterisks indicate significant differences and * represents *p* < 0.1. The unmarked represents “ns” (not significant). Statistical analysis was performed using the Student’s *t*-test.

Phenotypic identification was performed on transgenic and wild-type plants cultured for eight weeks (Figure 7A). The results showed that the height of overexpressed plants was 26% lower than that of wild-type plants, and the dry and fresh weights of aboveground parts were also reduced, but there was no significant difference in root length (Figure 7B–E). Both wild-type and transgenic plants grew better than the non-symbiotic plants under fungal inoculation. The growth of wild-type plants was better than that of transgenic plants, but there was no significant difference in the length of the primary root, as in non-symbiotic plants.

Based on the above statistical analysis, the overexpression of *ZmHPAT2* had an effect on plant height in non-symbiotic conditions, which inhibited plant height but had no effect on root length. Under symbiotic conditions, the growth of wild-type plants was better than that of transgenic plants. This is mainly because *ZmHPAT2* negatively regulates symbiosis with AM fungi during the symbiotic process.

We also statistically analyzed the symbiosis rates of AM fungi. Microscopic examination showed that both wild-type plants and overexpressing transgenic plants established symbiosis with AM fungi. The symbiotic rate of the two plants was calculated separately, and the results showed that the symbiotic rate of the overexpressed transgenic plants was slightly reduced compared to that of the wild-type plants, but it did not reach a significant level. In addition, the arbuscular in overexpressing transgenic plants was mostly smaller and not as full of whole cells as in the wild type (Figure 7F). In addition, the wild type also contained a large number of vesicles; on the contrary, no vesicle structure was observed in the overexpressed transgenic plants (Figure 7F,G). Based on this, we counted the arbuscule size, and the results showed that overexpression plants contained more shorter arbuscule less than 30um in length, whereas the arbuscules of 30–50 um were relatively fewer, consistent with the phenotype (Figure 7H).

### 2.8. ZmHPAT2 Regulates the Expression of the Auxin-Responsive Gene GH3 and the Branching Degradation-Related Gene ZmMYB1

**Figure 8:**
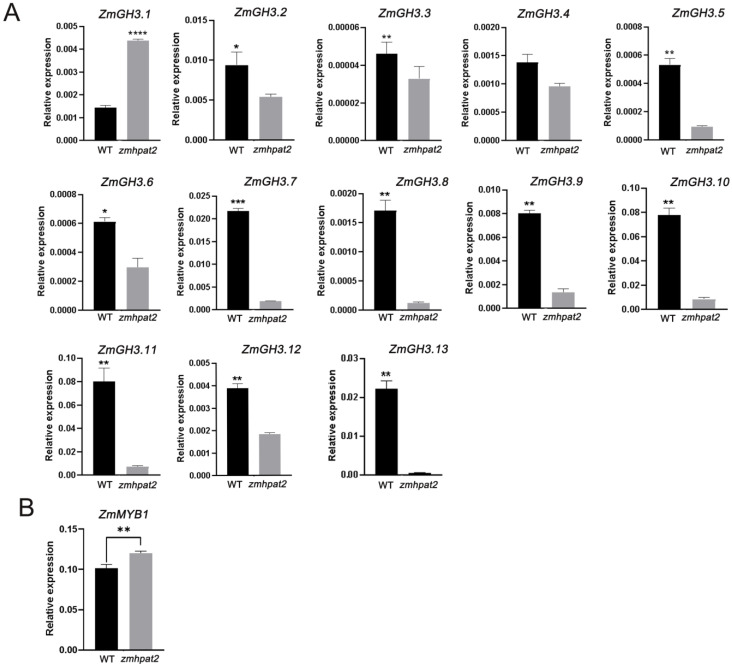
*ZmHPAT2* regulates the expression of related genes. (**A**) The relative expression of *ZmGH3S* genes in *zmhpat2* mutant maize. (**B**) Relative expression level of the arbuscule degradation gene *ZmMYB1* under AM fungal inoculation. Asterisks indicate significant differences and * represents *p* < 0.1, ** *p* < 0.01 and *** *p* < 0.001, **** *p* < 0.0001. The unmarked represents “ns” (not significant). Statistical analysis was performed using the Student’s *t*-test.

Studies have shown that HPAT family genes can modify small CLE peptides, which are involved in the polar transport of auxin in plants [22,29]. Since the root length of the mutant is shorter than that of the wild type under both symbiotic and non-symbiotic conditions, and the symbiotic rate of the mutant is higher than that of the wild type under symbiotic conditions, it is hypothesized that this may be due to the excessive accumulation of auxin in the roots. GH3 is a family of auxin response genes, which is one of the fastest responding gene families to auxin changes in plants, and maintains auxin homeostasis by conjugating excess free auxin in plants with amino acids [30]. Therefore, 13 genes of maize GH3 auxin response gene family were selected in this study [31], and qRT-PCR was used to detect the expression levels of each gene. The results showed that 12 out of 13 genes in the maize GH3 gene family were significantly downregulated in the mutant relative to the wild type (Figure 8A). Therefore, the expression of *ZmGH3*s gene is not inhibited in wild-type plants, and the auxin in maize can be negative feedback regulated to maintain auxin balance. The mutant *zmhpat2* negatively regulates the *ZmGH3*s gene, resulting in compromised inactivation of free auxin in vivo, thus it inhibited root elongation, while also promoting plant height to some extent. Furthermore, auxin can positively regulate the symbiosis between maize and AM fungi, and mainly affect the arbuscular abundance, which is similar to the previous statistical results.

As previously mentioned, fewer arbusculars were observed in the mutant *zmhpat2* than in the wild type at 60 days of symbiosis. In order to verify whether the reduction in arbuscular is related to the expression of arbuscular degradation related genes, the arbuscular degradation related gene *ZmMYB1* was selected for quantitative analysis, and it was found that the gene was more significantly expressed in the mutant *zmhpat2* (Figure 8B). The results indicated that the expression of *ZmMYB1* in mutant *zmhpat2* affected the degradation of arbuscular structure. After the mutation of *ZmHPAT2*, the expression of *ZmMYB1* is upregulated, resulting in more serious arbuscular degradation than that of the wild type, which is consistent with the results in statistical arbuscular structure. However, in overexpressed transgenic plants, the results were opposite to those in mutant plants, which further verified the role of *ZmHPAT2* in regulating arbuscular growth.

## 3. Discussion

Maize stands as one of the most widely consumed crops globally and serves as a major staple in China [32]. Germplasm innovation in maize has consistently been a central focus in agricultural research [33]. In recent years, CLE peptide hormones have emerged as a significant area of study, playing a pivotal role in various aspects of plant growth and development, including root formation, plant height regulation, and pollen development [34]. However, the effects of CLE peptides vary across different plant species. Despite their importance, research on CLE peptides in maize remains limited due to their diversity and susceptibility to inactivation in vitro. The hydroxyproline-arabinose transferase (HPAT) gene family, which is responsible for modifying CLE peptides, is essential for their functional activation [29]. Investigating the genes within this family can provide valuable insights into the roles of CLE peptide hormones. To date, most studies on HPAT have concentrated on model organisms such as *Arabidopsis*, tomato, and certain leguminous plants, leaving the functional analysis of HPAT genes in maize largely unexplored. Consequently, elucidating the functions of the HPAT gene family in maize holds significant potential for advancing maize research and enhancing germplasm resource development.

In this study, the evolutionary traits, structural characteristics, and functional roles of the *ZmHPAT2* gene were comprehensively investigated. The *ZmHPAT2* gene spans 1128 base pairs, encoding a protein of 375 amino acids, and is classified within the HPAT gene family. It features a highly conserved GT95 catalytic domain and is localized to the Golgi apparatus. Functionally, *ZmHPAT2* was found to significantly influence plant height, flowering time, and root development in maize. Specifically, the *zmhpat2* mutant exhibited a markedly increased plant height compared to the wild type, flowered 9 days earlier, and displayed a significantly shorter nodal root. Furthermore, heterologous overexpression of *ZmHPAT2* in *Lotus japonicus* resulted in a substantial reduction in plant height and a delay in flowering time. These findings underscore the critical role of *ZmHPAT2* in regulating key developmental processes in maize and highlight its potential functional conservation across species.

Recent studies have revealed that CLE peptides play a crucial role in regulating nodule formation in legumes and mediating symbiotic interactions between plants and microorganisms. In *Lotus japonicus*, the CLE peptide signals *LjCLE-RS1* and *LjCLE-RS2*, expressed in roots, are induced by rhizobia [35]. These peptides, particularly *LjCLE-RS2* and *LjCLE-RS1*, regulate nodule number by transmitting signals to the KLV receptor in the shoot [36,37]. Similarly, in peas, mutations impairing the CLE receptor *CLV2* homolog lead to increased nodulation [38]. In soybeans, *GmNARK*, a homolog of *CLV1*, has also been reported to control nodule number [39,40,41]. Additionally, in certain plants, root-derived CLE peptide signals can suppress nodule formation [42,43]. Phylogenetic analysis further identified the gene *PLENTY* as a direct homolog of *MtRDN1* [21]. Legumes have evolved a sophisticated mechanism, known as the autoregulation of nodulation (AON) pathway, to balance the energy demands of nodule formation for nitrogen fixation with other biological processes [44]. Genes such as *LjPLENTY*, *MtRDN3*, and *PsNOD12*, belonging to the HPAT gene family, mediate the arabinosylation of CLE peptides, thereby influencing AON in their respective species. These findings suggest that HPAT family genes modulate plant–rhizobia symbiosis through CLE peptide modification. However, the role of HPAT genes in maize and their interaction with symbiotic microorganisms remain unexplored.

To address this gap, this study identified the maize *ZmHPAT2* gene based on reported HPAT genes and investigated its role in symbiosis with arbuscular mycorrhizal (AM) fungi using mutant analysis. Promoter cis-acting element analysis revealed that the *ZmHPAT2* promoter contains multiple elements associated with symbiosis. Furthermore, hairy root transformation and GUS staining experiments demonstrated that *ZmHPAT2* expression is induced by AM fungi. In AM symbiosis experiments, the *zmhpat2* mutant exhibited a higher colonization rate than the wild type, suggesting that *ZmHPAT2* negatively regulates symbiosis. At 30 days post-inoculation, the mutant displayed a higher number of arbuscules compared to the wild type, potentially due to altered auxin activity. However, by 60 days, the arbuscule number in the mutant was significantly lower than in the wild type. Quantitative analysis of *ZmMYB1*, a gene involved in arbuscule degradation, revealed higher expression in the mutant at 60 days, indicating more severe arbuscule degradation. This explains the reduced arbuscule number in the mutant during later symbiotic stages. These results demonstrate that *ZmHPAT2* regulates arbuscule degradation in the later phases of symbiosis, highlighting the importance of the HPAT gene family in maize symbiosis. These findings align with previous studies in other species. For instance, in *Medicago sativa*, the *CLE53* gene is upregulated by AM fungi, and its RNAi-mediated silencing enhances AM symbiosis markers [45]. Additionally, the pretreatment of *Medicago sativa* seedlings with *RiCLE1* from *Rhizophagus irregularis* promotes mycorrhizal formation [46]. However, this study is the first to identify the symbiotic function of *ZmHPAT2* with AM fungi in maize and to clarify its role in regulating arbuscule degradation during later symbiotic stages. This discovery not only provides a new direction for maize research but also offers novel insights into the symbiotic mechanisms between maize and AM fungi.

Furthermore, studies utilizing EMS-induced mutant lines have demonstrated that *ZmHPAT2* significantly influences maize growth and development. The *zmhpat2* mutant exhibits shorter roots compared to the wild type, yet its plant height surpasses that of the wild type. Notably, the pollen release time of the *zmhpat2* mutant is 9 to 10 days earlier than that of the wild type. Previous research has shown that mutants of the receptor-like protein kinase 2 (*RPK2/TOAD2*) also display a short root phenotype, characterized by misoriented cell division in the root apical meristem (RAM) and insensitivity to *CLE17* and *CLE19* peptides, which inhibit root growth. This suggests that *RPK2/TOAD2* regulates RAM activity and primary root growth by recognizing *CLE17* and *CLE19* [47]. However, the relationship between HPAT family genes and root length remains unexplored. Similarly, researchers have identified a maize dwarf mutant, *d129*, through EMS induction. Phenotypic analysis revealed that the dwarfism in *d129* is caused by shortened internodes, accompanied by significantly reduced levels of IAA and GA3 compared to wild-type plants [48]. Additionally, maize mutants *d1*, *d3*, *d5*, *d10*, and *d11* all exhibit dwarfism and reduced internode length [49,50]. The maize *ZmABCB1* gene, encoding an ATP-binding auxin transporter, also shows reduced plant and ear height in its mutant [51]. These findings collectively highlight a strong correlation between auxin and plant height regulation.

In this study, we observed that 12 out of 13 auxin-responsive genes were significantly downregulated in the *zmhpat2* mutant. This suggests that *ZmHPAT2* may participate in auxin metabolic pathways and regulate plant development through auxin signaling, aligning with previously reported results. In rice, the CLE gene family member *FON2* controls floral development [52], while *FON2*-like CLE protein 1 (FCP1) [53] and *FON2 SPARE1* (*FOS1*) [54] regulate inflorescence size and floral meristem tissue. In tomatoes, mutations in CLV pathway components (*SlCLV1*, *SlCLV2*, *SlCLV3*, and *SlHPAT3*) lead to meristem expansion, resulting in clustered inflorescences and increased fruit set rates. Interestingly, treatment with arabinose-modified *SLCIV3* can restore normal meristem development in these mutants [55]. These studies provide partial explanations for the earlier pollen release observed in the *zmhpat2* mutant, suggesting that *ZmHPAT2* may regulate maize pollen development. However, further experiments are required to confirm this relationship. In summary, *ZmHPAT2* plays a multifaceted role in maize development, influencing root growth, plant height, and reproductive timing, likely through its involvement in auxin signaling and CLE peptide modification. These findings underscore the importance of *ZmHPAT2* in maize growth regulation and provide a foundation for future research into its molecular mechanisms.

Based on the findings outlined above, we propose a dual regulatory mechanism for *ZmHPAT2*. On the one hand, we hypothesize that *ZmHPAT2* modulates the expression of GH3 family genes. In the *zmhpat2* mutant, the downregulation of GH3 family genes results in elevated levels of free auxin. This auxin accumulation in the roots likely contributes to the observed shortening of root length in the mutant. Concurrently, the reduced expression of GH3 genes may also explain the increased plant height in the mutant, a phenomenon consistent with previous reports. For instance, silencing *GH3.8* in tomatoes has been shown to result in taller plants [56]. Following fungal inoculation, the high auxin concentration in the roots of the mutant may further enhance its symbiotic interaction with arbuscular mycorrhizal (AM) fungi. On the other hand, during the later stages of symbiosis, the upregulation of *ZmMYB1* genes in the *zmhpat2* mutant leads to more pronounced degradation of mycorrhizal structures. However, it remains unclear whether *ZmHPAT2* exerts its regulatory effects on these genes directly or indirectly through CLE peptides. This study did not explore this aspect in detail, and further experiments will be necessary to elucidate the underlying molecular mechanisms. In summary, *ZmHPAT2* appears to play a critical role in regulating auxin homeostasis and mycorrhizal symbiosis, potentially through its influence on GH3 and *ZmMYB1* gene expression. Future research should focus on clarifying the direct or indirect pathways through which *ZmHPAT2* mediates these effects, providing deeper insights into its functional role in maize development and symbiosis.

## 4. Materials and Methods

### 4.1. Plant Materials and Culture Substrates

The maize wild-type B73 inbred line and *L. japonicus* MG20 used in the study were obtained from the Key Laboratory of Crop Stress Resistance and High-Quality Biology at Anhui Agricultural University. The *zmhpat2* mutant of B73 background maize was screened from the maize EMS mutant library of Professor Lu Xiaoduo from Anhui Agricultural University. Thereafter, the mutant was backcrossed with B73 as the recurrent parent. Through successive backcrossing of mutant plants, followed by four generations of backcrossing, homozygous mutants were obtained via self-crossing for subsequent gene functional analysis. The method for identifying the *ZmHPAT2* homozygous mutant is to design specific amplification primers for approximately 1000 bp, with 4–500 bp upstream and downstream of the mutation site. After extracting the genomic DNA from the mutant plants and performing PCR amplification, PCR products with the desired bands are selected for sequencing. The sequencing results are then compared to determine whether a mutation has occurred at the mutation site, resulting in a stop gained of the amino acid sequence. The AM fungal species used was *Glomus intraradices*, provided by Sun Yat-sen University in Guangzhou, China.

The maize materials for the control and experimental groups used in the AM fungal inoculation experiment were cultivated in a substrate mixture of vermiculite, perlite, and river sand in a 6:1:1 ratio, which was sterilized at 121 °C in an autoclave for 40 min before use. The remaining soil-based maize experiments used a substrate mixture of Danish soil (Jutland, Denmark) and black soil (Liaoning, China) in a 3:1 ratio, which did not require sterilization. The substrate for cultivating *L. japonicus* was a mixture of vermiculite (Hefei, China) and perlite (Hefei, China) in a 3:1 ratio, which was sterilized at 121 °C in an autoclave (HIRAYAMA, Tokyo, Japan) for 40 min before use.

### 4.2. Plant Growth and AMF Inoculation

The experiment selected mold-free, plump, and size-consistent wild-type B73 seeds and EMS-induced *zmphat2* mutant seeds. The seed surfaces were disinfected by immersing them in a 12% sodium hypochlorite solution with 100 µL Tween, with continuous stirring for 10 min. Afterward, the seeds were rinsed with pure water, until the foam was completely washed away. The disinfected seeds were placed onto germination paper that had been sterilized with Captan (2.5 g of Captan dissolved in 1 L of pure water) for pre-germination. The seeds were then incubated in a growth chamber at 28 °C with 16 h of light and 6 h of darkness for germination. After germination, seeds with consistent growth were selected and transplanted into pots (35 cm × 24 cm) (Suqian, China) containing sterilized substrate. Before transplantation, the substrate was mixed with some AM fungal *Gi* spores, and a portion of sand spores were buried near the germinated seeds. On the day of transplantation, an appropriate number of liquid spores was added, with approximately 1000 spores per pot. After transplantation, the plants were placed in the greenhouse of the Agricultural Cultivation Garden at Anhui Agricultural University for cultivation.

### 4.3. RNA Isolation and qRT-PCR

Maize total RNA was isolated using the phenol–chloroform extraction method, and cDNA synthesis was performed using the HiScript II 1st Strand cDNA Synthesis Kit (Vazyme, Nanjing, China). qRT-PCR was performed using ChamQ Universal SYBR qPCR Master Mix (Vazyme, Nanjing, China) on a PikoReal Real-Time PCR System (TCR0096, Thermo Scientific, Waltham, MA, USA), following the manufacturer’s instructions. The expression of reference genes *Zmα-Tubulin* and *ZmGAPDH* was used as internal controls for maize. Relative gene expression was calculated using the 10^−(∆Ct/3)^ method based on three biological replicates per sample, with expression levels normalized to those of non-inoculated AMF maize [57].

### 4.4. Bioinformatics Analysis

To identify the homologous sequences of the *Arabidopsis* HPAT gene family in maize, a BLASTP homology search was conducted in the local maize protein database using the protein sequence, with an E-value less than 10^−70^. After retrieving the candidate proteins, the protein sequence with the highest homology was selected, and a BLASTP homology search was then performed in the *Arabidopsis* protein database, with an E-value less than 10^−70^. If *Arabidopsis* family gene can be screened, it is the homologous gene of HPAT in maize. The protein sequences of the identified homologous genes were analyzed using the online platforms PFAM (http://pfam.xfam.org/, accessed on 21 February 2023) and SMART (http://smart.embl-heidelberg.de/, accessed on 21 February 2023) to determine the conserved domains present in the proteins. The full sequence of *ZmHPAT2* was obtained from the Gramene website (https://ensembl.gramene.org/Zea_mays/Info/Index, accessed on 22 February 2023), and combined with the annotation information of the ZmHPAT2 gene sequence and CDS sequence from the maize genome annotation file to generate the gene structure diagram of ZmHPAT2. The protein sequences of the maize HPAT gene family and the reported HPAT genes from other species were aligned using MEGA7 (version 11.0.10). The alignment file was then output, and a phylogenetic tree was constructed in MEGA7 (version 11.0.10) using the Neighbor-Joining method. The upstream promoter sequence of approximately 1200 bp for *ZmHPAT2* was obtained from the Gramene website (https://www.gramene.org/Zeamays/Info/Index, accessed on 22 February 2023) and aligned with the maize chromosome data for verification. Subsequently, cis-acting elements related to symbiosis were found, and these data were submitted to RAST (https://rsat.eead.csic.es/plants/dna-pattern_form.cgi, accessed on 26 February 2023) for analysis of the cis-acting elements in the *ZmHPAT2* promoter, with a schematic diagram generated as output.

### 4.5. Subcellular Localization in Maize Protoplasts

The *ZmHPAT2*::GFP and 1305::GFP plasmids, along with marker plasmid, were co-transformed into maize protoplasts using PEG. After 16 h, the fluorescent signals of red fluorescent protein (RFP) and green fluorescent protein (GFP) were observed and captured using a confocal microscope (Leica LSM800, Wetzlar, Germany) with excitation wavelengths of 514 nm and 488 nm. The empty 1305::GFP plasmid was used as a control.

### 4.6. Mycorrhizal Staining and Symbiosis Rate Determination

The infection rate needs to be determined by using staining methods to facilitate the observation of symbiosis. The steps of fixation, clarification, acidification, staining, and decolorization are included in the staining procedure. First, the cleaned roots are cut into segments of approximately 2–3 cm in length and immersed in FAA fixative for more than 4 h in a fume hood. Next, rinse the root segments with distilled water to remove the fixative, then immerse the root segments in 10% KOH and heat them in a 90 °C water bath for an appropriate amount of time until the roots become transparent. The heating time should not be too long. Afterward, allow the root segments to cool to room temperature in the fume hood. Acidify the root segments with 5% lactic acid until they turn transparent white. Rinse the root segments with distilled water to remove the acid, then immerse them in either toluidine blue or fuchsin staining solution. Heat the root segments in a 90 °C water bath for approximately 30 min and then allow them to cool to room temperature in the fume hood. Finally, rinse the stained root segments with distilled water to remove the staining solution. Then, immerse all the root segments in lactoglycerol and shake them on a shaker at 120 rpm/min for decolorization. Replace the decolorization solution with fresh solution as it turns blue and continue until no further decolorization occurs. Afterward, prepare the stained root segments into sections, observe under a microscope, and record the results.

### 4.7. Induced Transformation of Hairy Roots of L. japonicus

The first step is to disinfect the seeds of *L. japonicus*, then use sandpaper to rub the seeds, removing the waxy layer on the surface. Next, clean the seeds with 75% alcohol for 30 s in a laminar flow cabinet, then disinfect them with 12% Kao and 0.1% Tween 20 for 10 min. During this process, the centrifuge tube should be constantly inverted and shaken to ensure the seeds are fully exposed to the disinfectant solution. Then, de-foam the seeds with 75% alcohol three times, each for 2 min. Finally, rinse the seeds with sterile water three times, each for 5 min. After disinfecting the seeds, place them in a 4 °C refrigerator for 12 h to undergo vernalization, then evenly spread them in a square dish containing 1.2% water agar. Place the seeds horizontally in a 23 °C dark incubator for 12 h, then vertically for 24 h. Until the seeds have sprouted roots about 1 cm long, proceed with Agrobacterium infection. Transfer the previously stored *Agrobacterium tumefaciens* LBA9402 containing the target plasmid onto YMB solid medium containing antibiotics and incubate in the dark at 28 °C for 48 h until use. Prepare the B&D (Broughton and Dilworth) solid medium, sterilize it, then add 100 µM of acetosyringone (AS), and pour it into square dishes placed at a slight incline. Cut off 1/3 of the thinner edge of the solid B&D medium, and make several vertical incisions on the remaining medium. Use a sterile scalpel to cut off approximately 2 mm of the germinated root tip. Then, use tweezers to pick up the cut tip and infect it with *Agrobacterium tumefaciens* LBA9402, which has been transformed with the target plasmid. The infected *L. japonicus* are placed into the pre-made incisions. Finally, seal the square dish with sealing film and medical tape to prevent contamination by unwanted microbes. First, place them in the dark at 28 °C for 24 h, then transfer to a 23 °C incubator (16/8 h light/dark cycle) to grow for about 3 weeks.

### 4.8. Symbiosis Between L. japonicus and AM Fungi, and GUS Staining

Root hairs of *L. japonicus* grown on B&D solid medium for about 3 weeks were selected. The root hairs with swollen and more branching roots were chosen for genomic validation to confirm whether they were positive plants. Transplant these positive plants into the aforementioned substrate. The experiment is divided into two groups: one group is inoculated with AM fungi, and the other group is not inoculated with AM fungi. They are cultivated for 8 weeks in a 25 °C greenhouse (16 h of light/8 h of darkness) and irrigated with a customized Hoagland nutrient solution. After the cultivation is complete, the root hairs of the *L. japonicus* are sampled, washed, and then immersed in GUS staining solution for staining. The samples are then treated in a dark environment at 37 °C in an incubator for 24 h. GUS formula: 0.05 g Trypan blue, lactic acid: glycerol: distilled water (1:1:1) 100 mL.

### 4.9. Statistical Analysis

The data were collected from three independent replicate experiments, analyzed with Excel 2019, and visualized using GraphPad Prism (version 8.0.2). Student’s *t*-test was used to determine significant differences, with * *p* ≤ 0.05 indicating a significant difference, ** *p* ≤ 0.01 indicating a highly significant difference, and “ns” indicating no significant difference.

## 5. Conclusions

The *ZmHPAT2* gene, with a coding region of 1128 bp, encodes a protein of 375 amino acids. It is located on maize chromosome 6 and contains the GT95 glycosyltransferase catalytic domain, which is localized to the Golgi apparatus. Promoter cis-acting element analysis revealed the presence of multiple symbiosis-related elements, suggesting a role in symbiotic interactions. Through symbiosis heatmap analysis, quantitative PCR, and *Lotus japonicus* root hair transformation experiments, it was demonstrated that *ZmHPAT2* expression is induced by arbuscular mycorrhizal (AM) fungi.

Studies using EMS-induced mutants revealed that *ZmHPAT2* significantly influences maize growth and development, as well as its symbiosis with AM fungi. The *zmhpat2* mutant exhibited shorter root systems compared to the wild type but displayed enhanced plant height. In terms of symbiosis, the *zmhpat2* mutant showed a higher symbiotic rate, characterized by increased arbuscule abundance during the mid-symbiotic phase and a greater number of vesicles during the late symbiotic phase. These findings suggest that *ZmHPAT2* may act as a negative regulator of symbiosis.

Quantitative analysis of the auxin-responsive *ZmGH3* gene family revealed that 12 out of 13 genes were significantly downregulated in the *zmhpat2* mutant. This indicates that *ZmHPAT2* likely participates in the auxin metabolic pathway, regulating plant development and fungal symbiosis through auxin signaling modulation. Additionally, quantitative analysis of the arbuscule degradation gene *ZmMYB1* showed that its expression was significantly upregulated in the *zmhpat2* mutant, consistent with the observed reduction in arbuscule numbers during the later symbiotic stages. This suggests that *ZmHPAT2* plays a role in regulating arbuscule degradation in the later phases of symbiosis.

In summary, *ZmHPAT2* is a key regulator of maize growth, development, and AM symbiosis, likely through its involvement in auxin signaling and arbuscule degradation pathways. These findings provide valuable insights into the molecular mechanisms underlying plant-fungal interactions and auxin-mediated developmental processes in maize.

## Data Availability

The original contributions presented in this study are included in the article/Supplementary Material. Further inquiries can be directed to the corresponding author.

## Supplementary Materials

The following supporting information can be downloaded at: https://www.mdpi.com/article/10.3390/plants14101438/s1, Figure S1: Map of *zmhpat2* mutation site; Figure S2: Analysis of *ZmHPAT2* relative expression.
