# Supplementary material for: ZmHPAT2 Regulates Maize Growth and Development and Mycorrhizal Symbiosis
Source: Plants (Basel). 2025 May 11;14(10):1438. doi: 10.3390/plants14101438 (PMC12115135; doi:10.3390/plants14101438)
Supplement: Supplementary file 1 [file plants-14-01438-s001.zip › plants-3563983--Supplementary.pdf]

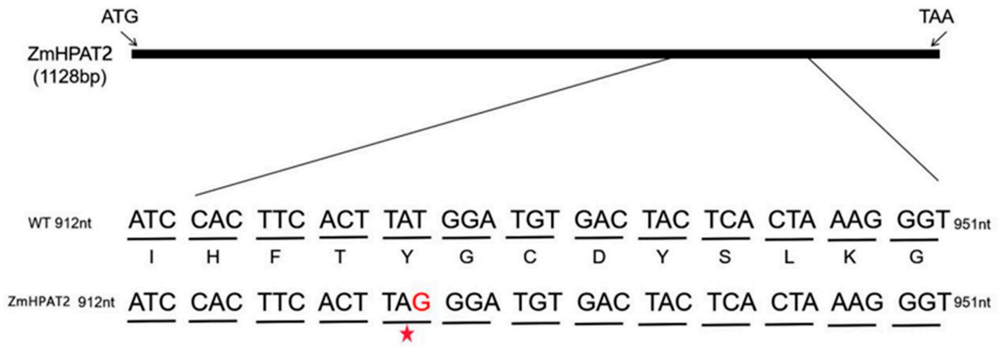

Figure S1. Map of *zmhpat2* mutation site

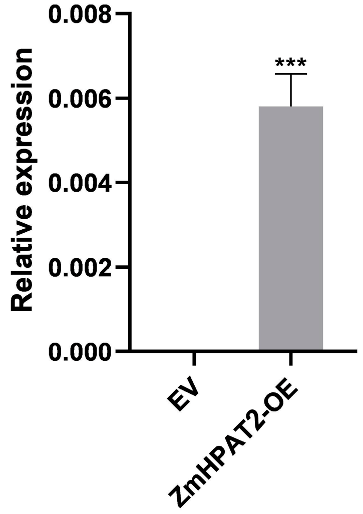

Figure S2. Analysis of *ZmHPAT2* relative expression. ***P<0.0001, Student's t-test.
